# Effects of Omega-3 Polyunsaturated Fatty Acid Supplementation on Non-Alcoholic Fatty Liver: A Systematic Review and Meta-Analysis

**DOI:** 10.3390/nu12092769

**Published:** 2020-09-11

**Authors:** Cheng-Han Lee, Yun Fu, Shih-Jyun Yang, Ching-Chi Chi

**Affiliations:** 1 Division of Gastroenterology and Hepatology, Department of Internal Medicine, Chang Gung Memorial Hospital, Linkou, Taoyuan 33305, Taiwan; george7467@hotmail.com; 2 Department of Dermatology, Chang Gung Memorial Hospital, Linkou, Taoyuan 33305, Taiwan; amyjoy791126@hotmail.com; 3 Department of Dermatology, Chang Gung Memorial Hospital, Keelung 20401, Taiwan; scanolqd@gmail.com; 4 College of Medicine, Chang Gung University, Taoyuan 33302, Taiwan

**Keywords:** fatty liver, omega-3 fatty liver, polyunsaturated fatty acid, steatohepatitis

## Abstract

(1) Aim: Non-alcoholic fatty liver disease (NAFLD) is a prevalent disease worldwide. Omega-3 polyunsaturated fatty acids (n-3 PUFAs) bear anti-inflammatory action and can ameliorate hyperlipidemia. We wish to appraise the effects of n-3 PUFAs supplement on NAFLD. (2) Methods: We searched CENTRAL, Embase, and MEDLINE on 29 March 2020 for randomized control trials (RCTs) on the effects of n-3 PUFAs supplementation in treating NAFLD. The Cochrane Collaboration’s tool was used to assess the risk of bias of included RCTs. (3) Results: We included 22 RCTs with 1366 participants. The risk of bias of included RCTs was generally low or unclear. n-3 PUFAs supplementation significantly reduced liver fat compared with placebo (pooled risk ratio 1.52; 95% confidence interval (CI) 1.09 to 2.13). n-3 PUFAs supplementation also significantly improved the levels of triglyceride, total cholesterol, high-density lipoprotein, and body-mass index, with pooled mean difference and 95% CI being −28.57 (−40.81 to −16.33), −7.82 (−14.86 to −0.79), 3.55 (1.38 to 5.73), and −0.46 (−0.84 to −0.08), respectively. (4) Conclusions: The current evidence supports the effects of n-3 PUFAs supplementation in improving fatty liver. n-3 PUFAs supplementation may also improve blood lipid levels and obesity.

## 1. Introduction

Non-alcoholic fatty liver disease (NAFLD) is getting attention globally on account of its increasing prevalence [1]. Among 1.5 billion people in a recent study, the primary type of chronic liver disease was NAFLD (60%), succeeded by hepatitis B (29%), hepatitis C (9%), and alcoholic liver disease (2%) [2]. The spectrum of NAFLD ranges from non-alcoholic fatty liver, which is defined as simple steatosis with no or scanty inflammation, to nonalcoholic steatohepatitis (NASH) [3], which may progress to fibrosis or even cirrhosis [4]. Liver cirrhosis is also considered end stage liver disease with presence of septal fibrosis and nodular parenchymal regeneration. Around 20% of NASH patients will develop cirrhosis over a lifetime [5]. NAFLD patients have increased mortality with increasing stages of liver fibrosis [6]. Approximately 33% of NASH patients present with advanced fibrosis and 20% may have regressed fibrosis during follow-up [7,8,9,10]. Previous evidence demonstrates associations of NAFLD with insulin resistance and type 2 diabetes mellitus (T2DM) [11,12]. Globally, 37.3% of T2DM patients suffer from NAFLD. [13]. Besides, several comorbidities including metabolic syndrome, cardiovascular disease, chronic kidney disease, and extrahepatic cancer have been reported [14,15].

Many factors involving genetic, metabolic, environmental, and gut microbial environment are linked to the development of NAFLD [15,16,17]. Though many studies have investigated the pathophysiology of NAFLD, the delicate mechanism remains obscure. Both hepatic and adipose tissue insulin resistance have been related to NAFLD. Systemic insulin resistance decreases plasma adiponectin and increases leptin concentrations. Insulin resistance also results in an excessive flux of fatty acid [18] as a result of unopposed adipose tissue lipolysis [19]. Thus, aggravation of fat accumulation in the liver may occur [17,19,20]. Because high levels of fasting insulin and associated hepatic insulin resistance are usually present in NAFLD patients, they are also susceptible to T2DM. Moreover, the pathogenesis of NAFLD also involves the immune system. Kupffer cells are specialized macrophages which regulate the immune response of the liver. Kupffer cells activation contributes to the formation of NASH and liver fibrosis [17,21,22,23].

Omega-3 polyunsaturated fatty acids (n-3 PUFAs) have been under heated discussion over the past two decades [24,25,26]. The n-3 PUFA family contain several long chain fatty acids, including α-linolenic acid (α-ALA), stearidonic acid (SDA), eicosapentaenoic acid (EPA), docosapentaenoic acid (DPA), and docosahexaenoic acid (DHA). Humans lack enzymes to introduce double bonds at the carboxyl end carbon beyond C-9 in fatty acid chain [24]. Hence, the n-3 PUFAs are so-called essential fatty acids as the human body cannot synthesize them de novo. The principal source of α-ALA is plants and it is derived mainly from seeds, nuts, and vegetable oil. SDA is a metabolic intermediate in the transformation of α-ALA to EPA and DHA [26,27]. The major n-3 PUFAs from the marine origin are EPA [26,27] and DHA in fish oils, while DPA is primarily derived from marine mammals. EPA and DHA effectively decrease the levels of triglyceride (TG) and very low-density lipoproteins (VLDL), which convert to low-density lipoprotein (LDL) and intermediate-density lipoprotein (IDL) [28,29]. Thus, n-3 PUFAs have been considered beneficial in preventing coronary artery disease [30].

Some studies proposed that dietary n-3 PUFAs ameliorate insulin resistance by regulating mitochondrial function or mediating anti-inflammatory effects [31,32]. Currently, the principal therapy for NAFLD is diet and lifestyle modifications to lose body weight, while improving liver steatosis and inflammation [33,34]. Pharmacotherapy is reserved for NASH patients, particularly with significant fibrosis. Pioglitazone or vitamin E or their combination therapy may be used in NASH, however no specific drugs are firmly recommended. For this reason, a systemic review and meta-analysis of marine n-3 PUFAs supplementation in NAFLD patients was conducted. The purpose of our research was to assess the effects of marine n-3 PUFAs by evaluating several aspects including hepatic steatosis, liver function, lipid profile, insulin resistance, anthropometric measurements, and safety.

## 2. Materials and Methods

We executed a systematic review and meta-analysis of randomized controlled trials (RCTs) on the efficacy and safety of marine n-3 PUFAs supplement in treating NAFLD. The Preferred Reporting Items for Systemic Reviews and Meta-Analyses [35] guidelines were followed in the reporting of the current study.

### 2.1. Data Sources and Literature Search Strategy

The Cochrane Central Register of Controlled Trials, MEDLINE, and Embase databases were searched from inception to 29 March 2020 for relevant trials. Our search strategy is presented in Table 1. No language or geographic restrictions were applied.

### 2.2. Study Selection 

We included studies that satisfied the inclusion criteria as follows: (1) RCT study design; (2) the participants were subjects with NAFLD; (3) the study intervention was marine n-3 PUFAs or fish oil supplement, while the comparator was placebo or another therapy. (4) If there were more than two groups with different therapeutic doses of n-3 PUFAs in one trial, we included the highest dose group in the analysis. Two authors (C.-H.L. and Y.F.) independently scanned the titles and abstracts and evaluated their eligibility. Discrepancies were settled by seeking the opinions of a senior author (C.-C.C.). 

### 2.3. Data Extraction 

One author (C.-H.L.) extracted the data including surname of first author, numbers of participants, publication year, country, and outcome data of the intervention group and control group from the included trials. Our outcomes of interest included: (1) improvement of steatosis assessed via ultrasonography and/or histology; (2) the severity of steatosis detected by magnetic resonance imaging-proton density fat fraction (MRI-PDFF); (3) biochemical markers, such as liver function test [plasma alanine aminotransferase (ALT), aspartate aminotransferase (AST), and gamma-glutamyl transferase (GGT)] and lipid profiles [total cholesterol (TC), TG, high-density lipoprotein cholesterol (HDL), and LDL]; (4) the severity of insulin resistance measured by homeostatic model assessment for insulin resistance score (HOMA-IR) and fasting blood sugar (FBS) levels; (5) anthropometric parameters such as obesity estimated by body mass index (BMI); and (6) adverse events (AEs). If the articles did not provide adequate data for analysis, we contacted the trialists to ask for detailed information. In studies that did not provide a standard deviation for change from baseline in continuous variables, a correlation coefficient of 0.5 was applied for imputation [36,37]. Another author (C.-C.C.) checked these data.

### 2.4. Risk of Bias Assessment

The Cochrane’s tool [38] was employed to evaluate the risk of bias of the included trials by two authors (C.-H.L. and Y.F.), while a third author (C.-C.C.) was responsible for confirming the judgement. Seven domains were judged as high, unclear, or low risk of bias in the RCTs: allocation concealment, blinding of participants and personnel, random sequence generation, selective reporting, blinding of outcome assessors, incomplete outcome data, and others biases. If a study did not offer the data on AEs, it would have been rated with unclear risk of selective reporting bias.

### 2.5. Statistical Analysis 

All analyses were executed by utilizing the Review Manager software, version 5.4 (The Nordic Cochrane Centre, The Cochrane Collaboration, 2020). We anticipated clinical heterogeneity and thus chose the random-effects model. Dichotomous variables were presented as risk ratio (RR) with 95% confidence interval (CI). Continuous variables were presented as mean difference [39] or standardized mean difference (SMD) with 95% CI when different scales were used to measure the same outcome. The statistical heterogeneity among different studies was measured by calculating the I^2^ index. An I^2^ of greater than 50% was regarded as moderate heterogeneity [34]. When the *p* was < 0.05, it was defined as statistical significance. We also conducted a subgroup analysis on numbers of participants with improvement of steatosis measured by different methods including ultrasonography and histology. 

## 3. Results

### 3.1. Characteristics of Included Studies 

**Figure 1:**
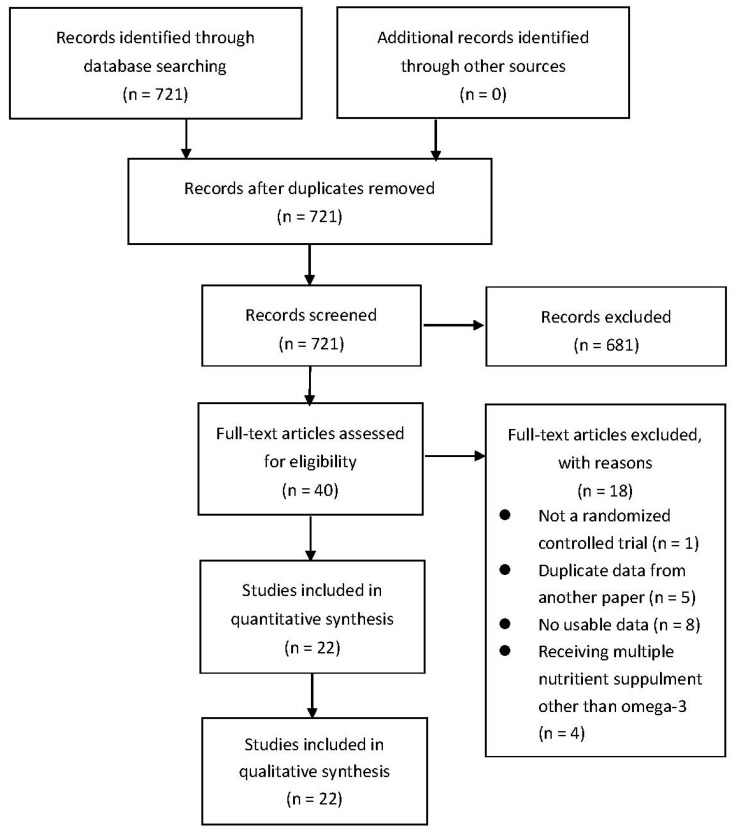
PRISMA Study Flow Chart.

Our search identified 721 publications after eliminating duplicates that the Preferred Reporting Items for Systematic Reviews and Meta-Analyses (PRISMA) flowchart is presented in Figure 1. After our initial screening by the titles and abstracts, 699 records were excluded. In the wake of inspecting the full text, 22 RCTs with 1366 participants were included. The characteristics of the included RCTs studies [3,40,41,42,43,44,45,46,47,48,49,50,51,52,53,54,55,56,57,58,59,60] are listed in Table 2. 

**Figure 2:**
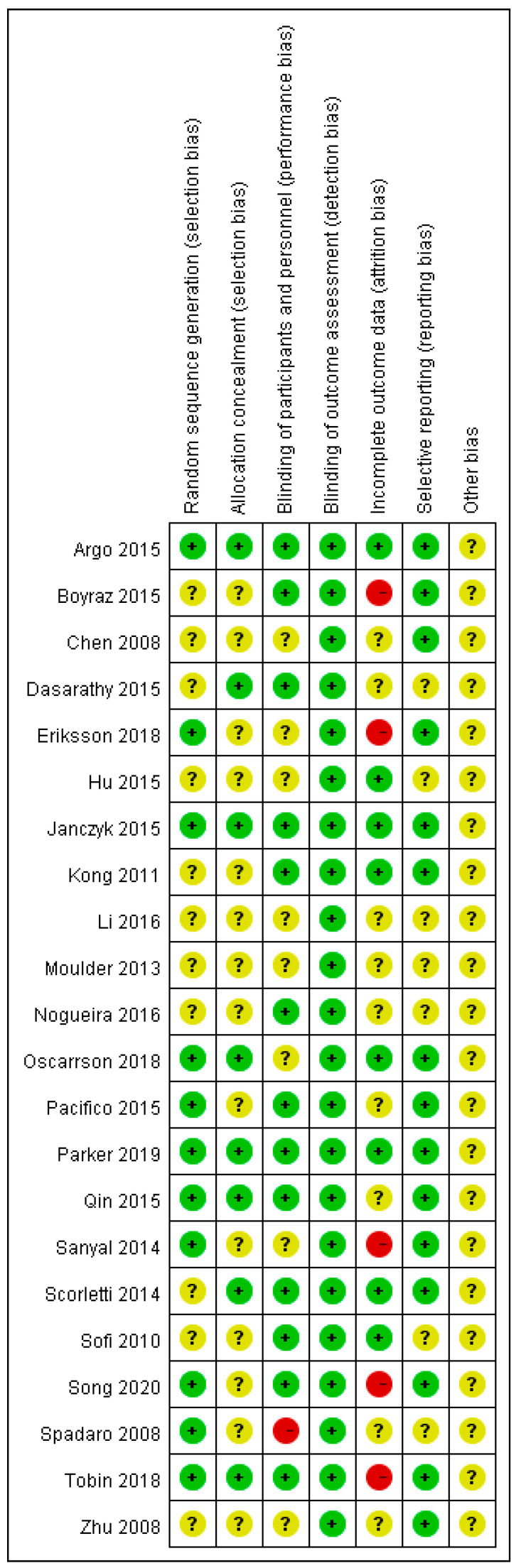
Risk of bias assessment of included trials. Green dots represent low risk of bias, with yellow and red for unclear and high risk of bias, respectively.

We delineated the risk of bias judgement in Figure 2. Some of the included RCTs that did not sufficiently depict the details of random number generation, concealment of allocation, and the process of blinding for outcome assessors, which were rated with unclear risk of bias, were only published in the abstract. However, most included RCTs were judged as low or unclear risk in the items, with only few rated high risk.

### 3.2. Effect of Omega-3 Polyunsaturated Fatty Acids (n-3 PUFAs) on Liver Fat and Histology

**Figure 3:**
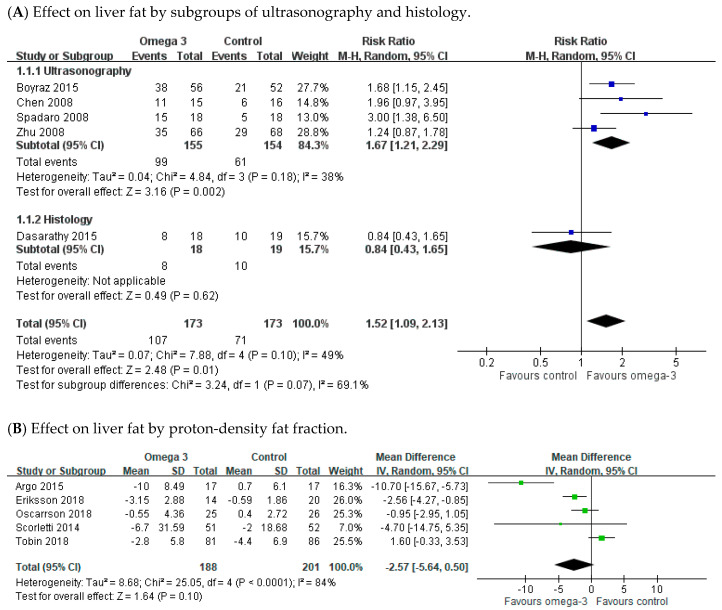

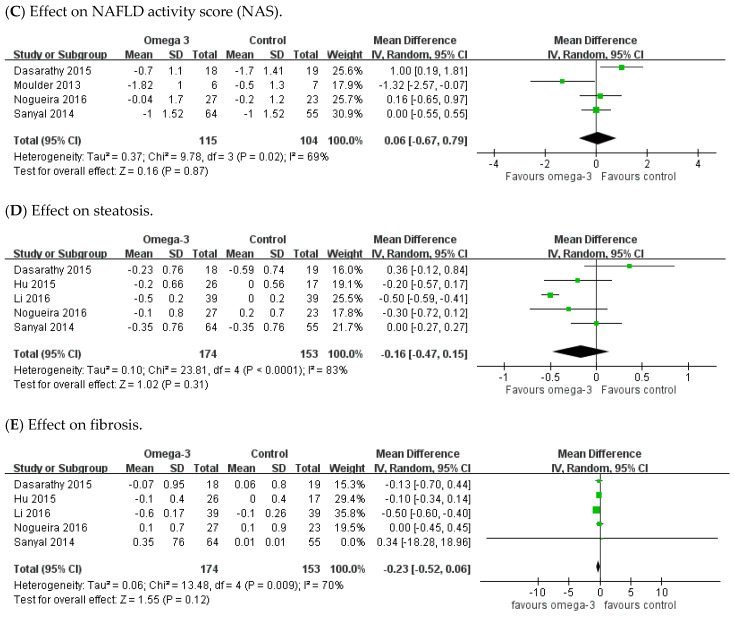
Effects of omega-3 fatty acid supplementation on liver fat and histology. (**A**) Effect on liver fat by sonography and histology. (**B**) Effect by proton-density fat fraction. (**C**) Effect on NAFLD score (NAS). (**D**) Effect on steatosis. (**E**) Effect on steatosis.

There were five RCTs illustrating the number of participants in the liver steatosis improvement among the n-3 PUFAs and control groups. NAFLD patients receiving n-3 PUFAs supplementation more frequently achieved improvement in liver fat compared with the placebo-treated patients (pooled RR 1.52; 95% CI 1.09 to 2.13). Of these five trials, steatosis was measured by ultrasonography in four RCTs, while only one RCT evaluated steatosis by histology (Figure 3A). A subgroup analysis showed greater improvement in liver steatosis by ultrasonography in the n-3 PUFAs group than controls (pooled RR, 1.67; 95% CI: 1.21 to 2.29); while on histology, no difference in liver fat improvement was noted between the n-3 PUFAs and placebo groups (RR 0.84; 95% CI 0.43 to 1.65).

There were five RCTs investigating the severity of steatosis via MRI-PDFF, with considerable heterogeneity among them (I^2^ = 84%). The meta-analysis revealed that the n-3 PUFAs group was more likely to reach amelioration in PDFF compared with the placebo group; however, it did not reach significant difference (Figure 3B). The pooled mean difference (MD) was −2.57 (95% CI −5.64 to 0.50). Four studies reported measurements of NAFLD activity score (NAS) in the n-3 PUFAs group and controls. There was moderate heterogeneity across these trials (I^2^ = 69%). The meta-analysis showed no difference in NAS between the n-3 PUFAs and control groups (Figure 3C). The pooled MD was 0.06 (95% CI −0.67 to 0.79). 

We also analyzed the change in steatosis and fibrosis separately under histology. There were five RCTs reporting the steatosis and fibrosis alteration between the n-3 supplement and control groups. There was remarkable heterogeneity across these trials (steatosis: I^2^ = 83%; fibrosis: I^2^ = 70%). Although, the meta-analysis revealed improvement of steatosis and fibrosis in the n-3 PUFAs supplement group, however without significant difference (Figure 3D,E). The pooled MD was −0.16 (95% CI −0.47 to 0.15) in steatosis and −0.23 (95% CI −0.52 to 0.06) in fibrosis.

### 3.3. Effect of n-3 PUFAs on Hepatic Enzyme Parameters

**Figure 4:**
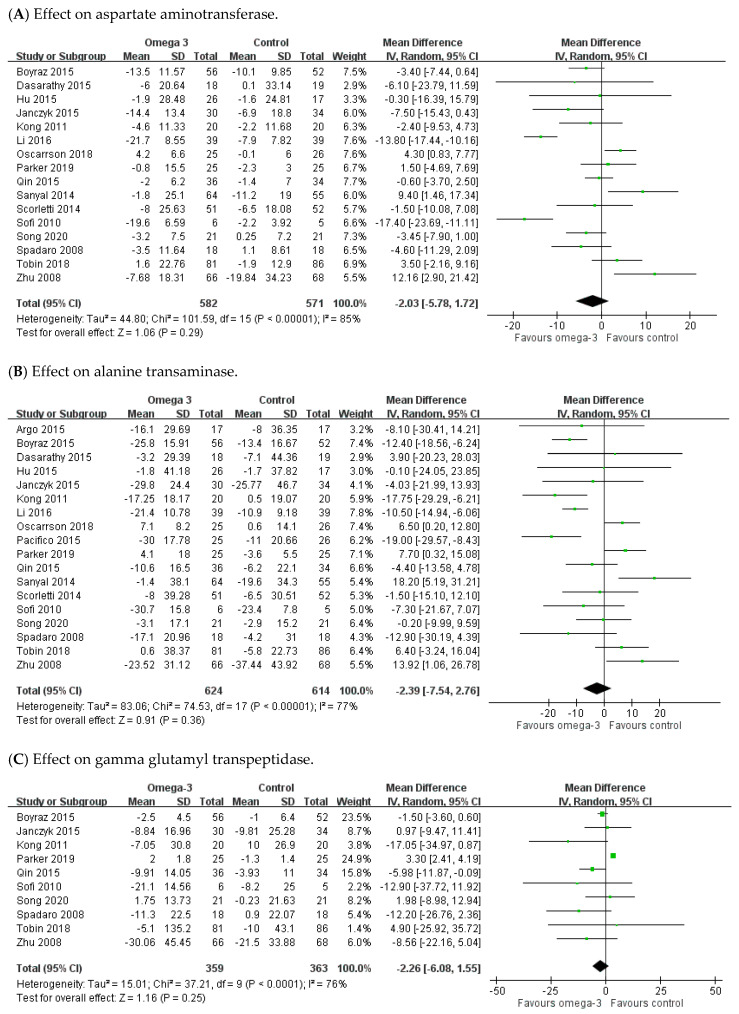
Effect of omega-3 fatty acid supplementation on hepatic enzyme parameters. (**A**) Effect on aspartate aminotransferase. (**B**) Effect on alanine transaminase. (**C**) Effect on gamma glutamyl transpeptidase.

In the analysis on hepatic enzymes, 16, 18, and 10 RCTs reported data on AST, ALT, and GGT, respectively. Statistical heterogeneity was shown among these studies (AST: I^2^ = 85%; ALT: I^2^ = 77%; GGT: I^2^ = 76%). The meta-analysis demonstrated a non-significant trend for improvement in hepatic enzymes in the n-3 PUFAs group when compared with controls. The pooled MD was −2.03 (95% CI: −5.78 to 1.72) for AST, −2.39 (95% CI: −7.54 to 2.76) for ALT, and −2.26 (95% CI: −6.08 to 1.55) for GGT, respectively (Figure 4A–C).

### 3.4. Effect of n-3 PUFAs on Serum Lipid Profiles

**Figure 5:**
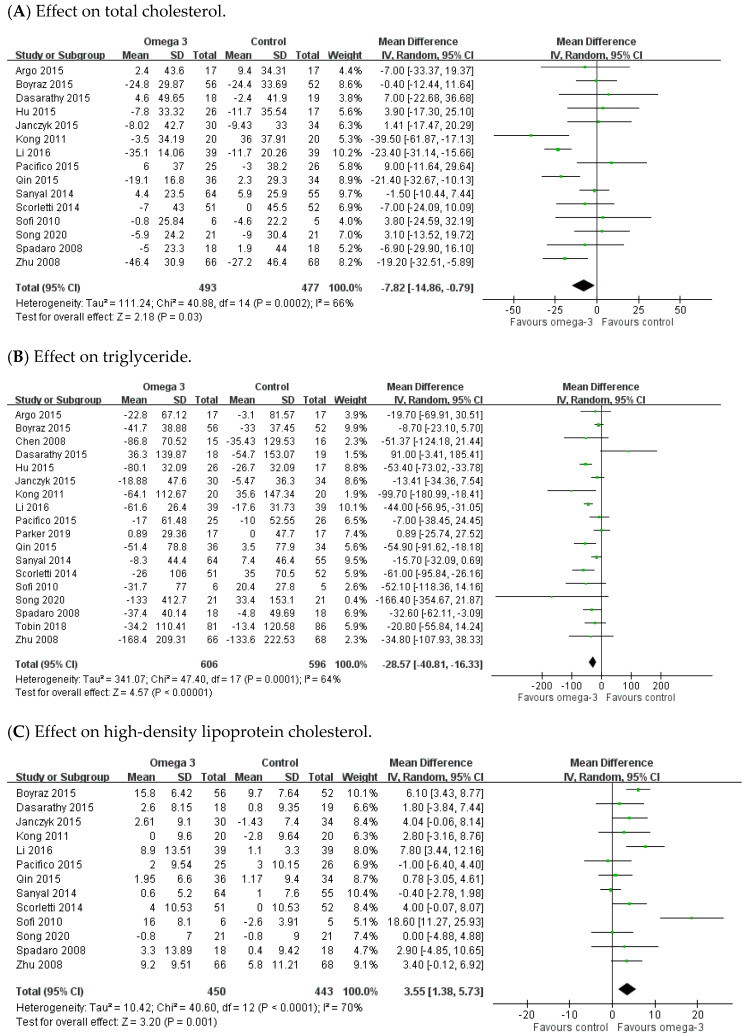

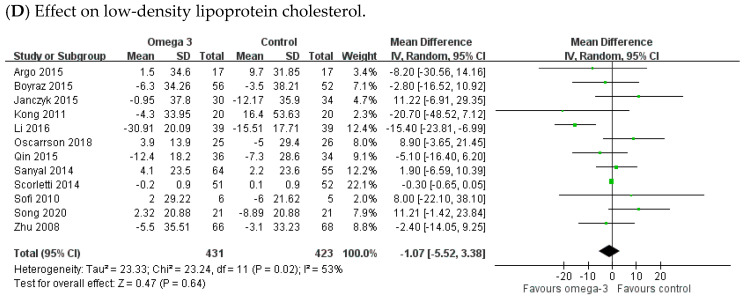
Effects of omega-3 fatty acid on serum lipid profiles. (**A**) Effect on total cholesterol. (**B**) Effect on triglyceride. (**C**) Effect on high-density lipoprotein cholesterol. (**D**) Effect on low-density lipoprotein cholesterol.

There were 18, 15, 13, and 12 RCTs providing data for TG, TC, HDL, and LDL, respectively. As for TG and TC domains, significant heterogeneity existed among these studies (TG: I^2^ = 64%; TC: I^2^ = 66%). The meta-analysis demonstrated a remarkable decrease of TG and TC in the n-3 PUFAs group, with the pooled MDs being −28.57 (95% CI: −40.81 to −16.33) and −7.82 (95% CI: −14.86 to −0.79), respectively (Figure 5A,B). As for HDL and LDL domains, there was significant heterogeneity across the trials (HDL: I^2^ = 70%; LDL: I^2^ = 53%). The meta-analysis demonstrated remarkable elevation of HDL (MD 3.55, 95% CI: 1.38 to 5.73), but no significant change in LDL (MD −1.07, 95% CI −5.52 to 3.38) associated with n-3 PUFAs supplement when compared to placebo (Figure 5C,D).

### 3.5. Effect of n-3 PUFAs on Fasting Blood Sugar and Homeostatic Model Assessment for Insulin Resistance

**Figure 6:**
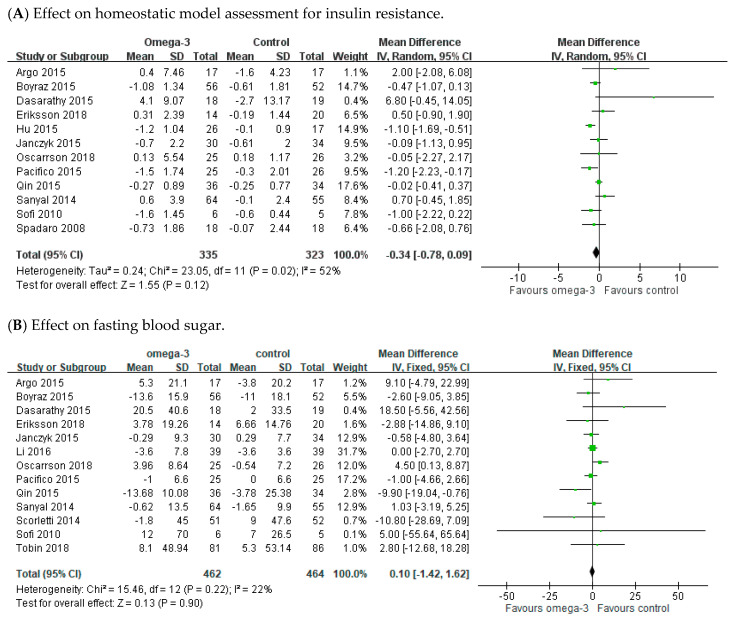
Effect of omega-3 fatty acid on fasting blood sugar and HOMA-IR. (**A**) Effect on homeostatic model assessment for insulin resistance. (**B**) Effect on fasting blood sugar.

There were 13 and 12 RCTs reporting measurement of FBS and HOMA-IR values, respectively. Moderate heterogeneity was detected across studies on HOMA-IR (I^2^ = 52%), but not on FBS (I^2^ = 22%). The meta-analysis demonstrated that n-3 PUFAs supplement did not significantly improve FBS and HOMA-IR. The pooled MD was −0.34 (95% CI: −0.78 to 0.09) in HOMA-IR (Figure 6A) and 0.1 (95% CI: −1.42 to 1.62) in FBS (Figure 6B).

### 3.6. Effect of n-3 PUFAs on Body Mass Index

**Figure 7:**
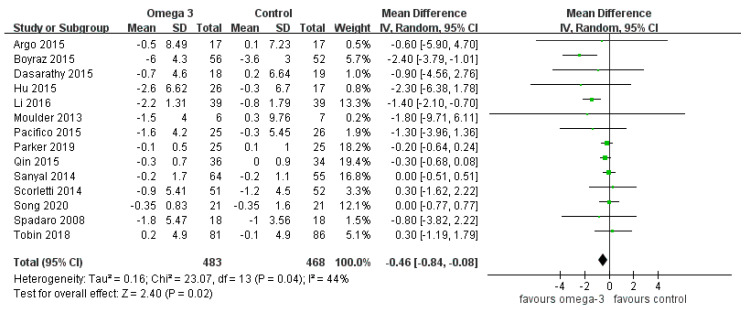
Effect of omega-3 fatty acid on body mass index.

There were 14 RCTs providing data on BMI. There was no significant heterogeneity (I^2^ = 44%). The meta-analysis demonstrated a greater decrease of BMI in the n-3 PUFAs group than the control (MD −0.46, 95% CI −0.84 to −0.08) (see Figure 7).

### 3.7. Adverse Events

There were 15 RCTs reporting AEs following n-3 PUFAs supplementation. The reported AEs were mild or moderate and generally well tolerated by patients, with nausea, mild abdominal discomfort, increased fecal frequency, epigastria, and defecation being reported. No serious adverse events occurred among the included RCTs. However, the treatment periods in the included studies were all shorter than two years.

## 4. Discussion

Our study is an updated systematic review and meta-analysis of RCTs on the effects of n-3 PUFAs supplement in treating NAFLD. In the present study, the liver fat in the n-3 PUFAs group was markedly improved compared to the control group, especially when detected by abdominal ultrasonography. PDFF, which was measured by MRI in five RCTs, was also more likely to decrease in the n-3 PUFAs group than the placebo group; however, no significant difference was detected between the two groups (MD −2.57; 95% CI −5.64 to 0.50).

Clinically, evidence of imaging or histology is needed for diagnosis of NAFLD. Ultrasonography is the most popular and is available globally with good sensitivity (84.8%) and specificity (93.6%) [61]. Positive findings include hyperechogenicity of the liver parenchymal tissue, brighter liver in contrast to the spleen and kidney, and blurring of vascular margins. Abdominal ultrasonography cannot detect trivial hepatic steatosis and cannot easily distinguish simple steatosis, NASH, or hepatic fibrosis [62]. Since histology is the golden standard of NAFLD diagnosis, liver biopsy is an invasive procedure with potential related complications. However, a liver biopsy could only obtain specimens of about 1/50,000 of the liver, of which accuracy may be reduced due to sampling error [63,64]. As for a histological perspective in our meta-analysis, there were no differences between both groups in NAS, fibrosis, and steatosis. In 15 of the 22 RCTs, the treatment period was ≤ 6 months (median: 6 months; range: 3 to 18 months). Histologically, the steatosis and fibrosis may take a longer period to achieve remarkable improvement; while in an animal study, a 3-week period of n-3 PUFAs supplement improved liver fat [65]. To the best of our knowledge, this meta-analysis is the first to examine the benefit of n-3 PUFAs on liver fat by different kinds of measurements. Hence, RCTs with a longer n-3 PUFAs treatment course and follow-up period may be warranted for seeing the effect on tissue fat or fibrosis.

As shown in Figure 3, five RCTs (Boyraz, 2015; Spadaro 2008; Argo 2015; Eriksson 2018; Li 2016) showed benefit of n-3 PUFAs on hepatic steatosis either by image or tissue proof [40,41,43,45,56]. Except for the Eriksson 2018 trial, which did not provide data of liver enzymes and lipid profile, we could see that the participants in the n-3 PUFAs supplementation group among the four other RCTs have significant amelioration or a trend for improvement in liver enzymes, lipid profile, and BMI. However, there is no consistent improvement in FBS or HOMA-IR among these RCTs. According to Li et al., people receiving n-3 PUFAs supplementation for 6 months had significant improvement in histological steatosis, liver enzymes, lipid profile, and BMI compared to the control group (normal saline). In that study, both groups were instructed to perform modest physical exercise for ≥ 5 days per week, while low-fat, cholesterol, and carbohydrate diets were given. The quality and quantity of exercise and diet might have affected the result of the studies. Thus, standardized food intake and exercise protocols should be employed in future trials.

Two RCTs that showed improvement in hepatic steatosis (Boyraz 2015, Spadaro 2008) were rated as having a high risk of bias for multiple reasons [41,56]. The Boyraz et al. study recruited 138 adolescents, however only 108 completed the protocol; so we rated it as high attrition risk. However, no obvious adverse effect was described with the protocol. The Spadaro 2008 trial was rated with a high risk of performance bias because the experimental group received PUFAs capsules twice a day, but the control group only received a dietary treatment. However, all the abdominal ultrasonography was performed by an operator blinded to the treatment allocation of the participants.

In the biochemical data of liver enzymes and metabolic status, we found significant improvement of TC, TG, HDL, and BMI. However, n-3 PUFAs supplementation did not show a remarkable benefit for AST, ALT, GGT, LDL, HOMA-IR, or FBS. Compared with the earliest systemic review of n-3 PUFAs supplement on NAFLD, similar results that there was a significant benefit for hepatic fat improvement and a trend of AST, ALT improvement were shown in the RCTs [66]. In respect of liver enzymes, our investigation showed a trend towards favoring n-3 PUFAs treatment on AST, ALT, and GGT. This is in accordance with the previous meta-analysis of RCT made by Yu et al., except ALT. However, the ALT effect in the previous meta-analysis revealed low heterogeneity and a fixed-effect model was applied [67]. In previous investigations, a high ratio of an omega-6/omega-3 diet in NAFLD patients may cause lipid proliferation and lipogenesis, which may trigger hepatosteatosis and further inflammation [18,66,68]. As for the FBS level and HOMA-IR, there was a different consequence between our study and a former review [18]. Imamura et al. reported that PUFAs could significantly ameliorate sugar level, hemoglobin A1C, and HOMA-IR compared to saturated fat. According to the study, the metabolic effect could be associated with omega-6, total PUFAs (mixed omega-3/omega-6), and not omega-3 alone. This result could explain, to some extent in our research, why the metabolic status did not show a remarkable effect because the placebo was omega-6 in some RCTs. However, the treatment dose and duration could not be described in the study. Compared to the previous research, our systematic review included seven more RCTs and provides the most updated research evidence [43,44,46,48,50,55,57].

n-3 PUFAs help cell membrane phospholipid fatty acid composition alteration, restriction of nuclear factor kappa B (a pro-inflammatory transcription factor), and activation of the anti-inflammatory transcription factor NR1C3 that may be achieved in the reduction of chronic diseases [69,70]. In metabolic aspects, n-3 PUFAs help to lower the plasma levels of TG, particularly in hypertriglyceridemia by inhibiting TC, TG, and VLDL synthesis in the liver. In one previous study [71], the recommended dose of n-3 PUFAs as an effective and safe option for TG reduction is > 3 g/day. In the present meta-analysis, we found that n-3 PUFAs supplement significantly improves the plasma levels of TC, TG, and HDL as well as the BMI in patients with fatty liver. There are 15, 18, 13, and 14 included studies with sufficient data of TC, TG, HDL, and BMI, respectively. In 60% (9/15), 89% (16/18), 77% (10/13), and 71% (10/14) of the included RCTs, the n-3 PUFAs group significantly improved in TC, TG, HDL, and BMI when compared to the controls. In most of these trials (TC: 89% (8/9), TG: 88% (14/16), HDL: 90% (9/10); BMI: 80% (8/10)), the treatment course was at least 6 months.

There were some limitations in our study. First, heterogeneity was observed among the RCTs, which might have been related to differences in ethnicity, ages (only children and adolescents were included in two studies), and sex (only male patients were included in one study). Second, although 22 RCTs were included, the sample size was generally small, with less than 30 in each group in 14 of the included studies. Only 5 RCTs could offer data on post-treatment histology. Third, there were various treatment doses, durations, and even regimens of therapy. Further research on the dose-response of n-3 PUFAs for fatty liver is warranted.

## 5. Conclusions

The current evidence supports the benefit of n-3 PUFAs supplementation in improving liver fat, especially on ultrasonography. n-3 PUFAs supplementation may improve the plasma levels of TC, TG, and HDL as well as BMI. Future RCTs with a large population and adequate length of outcome tracing are warranted to confirm the benefit and safety of n-3 PUFAs supplementation in treating NAFLD.

## Figures and Tables

**Table 1:** Search strategy.

Database	Search Strategy
Cochrane Central Register of Controlled Trials	#1 MeSH descriptor: [Fatty Liver] explode all trees #2 (“nonalcoholic fatty liver disease”):ti,ab,kw (Word variations have been searched) #3 (NAFLD):ti,ab,kw (Word variations have been searched) #4 (liver and (fatty or steatosis or steatoses)) #5 #1 or #2 or #3 or #4 #6 MeSH descriptor: [Fish Oils] explode all trees #7 Fish Oil:ti,ab,kw (Word variations have been searched) #8 fish liver oil:ti,ab,kw (Word variations have been searched) #9 MeSH descriptor: [Cod Liver Oil] explode all trees #10 Cod Liver Oil:ti,ab,kw (Word variations have been searched) #11 MeSH descriptor: [Fatty Acids, Omega-3] explode all trees #12 Omega-3:ti,ab,kw (Word variations have been searched) #13 Omega 3:ti,ab,kw (Word variations have been searched) #14 MeSH descriptor: [Eicosapentaenoic Acid] explode all trees #15 EPA:ti,ab,kw (Word variations have been searched) #16 eicosapentaenoic acid:ti,ab,kw (Word variations have been searched) #17 eicosapentaenoate:ti,ab,kw (Word variations have been searched) #18 icosapentaenoic acid:ti,ab,kw (Word variations have been searched) #19 MeSH descriptor: [Docosahexaenoic Acids] explode all trees #20 DHA:ti,ab,kw (Word variations have been searched) #21 docosahexaenoic acid:ti,ab,kw (Word variations have been searched) #22 docosahexaenoate:ti,ab,kw (Word variations have been searched) #23 #6 or #7 or #8 or #9 or #10 or #11 or #12 or #13 or #14 or #15 or #16 or #17 or #18 or #19 or #20 or #21 or #22 #24 #5 and #23
Embase	#1 ‘nonalcoholic fatty liver’/exp #2 ‘nonalcoholic fatty liver’:ti,ab #3 ‘NAFLD’:ti,ab #4 liver:ti,ab AND (fatty:ti,ab OR steatosis:ti,ab OR steatoses:ti,ab) #5 #1 OR #2 OR #3 OR #4 #6 ‘fish oil’:ti,ab #7 ‘fish liver oils’:ti,ab #8 ‘cod liver oil’:ti,ab #9 ‘omega 3 fatty acid’:ti,ab #10 ‘eicosapentaenoic acid’:ti,ab #11 ‘icosapentaenoic acid’:ti,ab #12 eicosapentaenoate:ti,ab #13 ‘docosahexaenoic acid’:ti,ab #14 docosahexaenoate:ti,ab #15 #6 OR #7 OR #8 OR #9 OR #10 OR #11 OR #12 OR #13 OR #14 #16 ‘crossover procedure’/exp #17 ‘double-blind procedure’/exp #18 ‘single-blind procedure’/exp #19 crossover*:ti,ab #20 ‘cross over’:ti,ab #21 placebo:ab,ti #22 double NEAR/3 blind #23 allocat*:ti,ab #24 trial:ti #25 ‘randomized controlled trial’/exp #26 random *:ti,ab #27 #16 OR #17 OR #18 OR #19 OR #20 OR #21 OR #22 OR #23 OR #24 OR #25 OR #26 #28 ‘animal’/exp OR ‘invertebrate’/exp OR ‘animal experiment’/exp OR ‘animal model’/exp OR ‘animal tissue’/exp OR ‘animal cell’/exp OR ‘nonhuman’/exp #29 ‘human’/exp OR ‘normal human’/exp #30 #28 AND #29 #31 #28 NOT #30 #32 #27 NOT #31 #33 #5 AND #15 AND #32
MEDLINE	1 exp Fatty Liver/ 2 nonalcoholic fatty liver disease.ti,ab. 3 NAFLD.ti,ab. 4 (liver and (fatty or steatosis or steatoses)).ti,ab. 5 or/1-4 6 exp Fish Oils/ 7 fish oil.ti,ab. 8 fish liver oil.ti,ab. 9 exp Cod Liver Oil/ 10 cod liver oil.ti,ab. 11 exp Fatty Acids, Omega-3/ 12 omega-3.ti,ab. 13 omega3.tu,ab. 14 exp Eicosapentaenoic Acid/ 15 EPA.ti,ab. 16 eicosapentaenoic acid.ti,ab. 17 eicosapentaenoate.ti,ab. 18 icosapentaenoic acid.ti,ab. 19 exp Docosahexaenoic Acids/ 20 DHA.ti,ab. 21 docosahexaenoic acid.ti,ab. 22 docosahexaenoate.ti,ab. 23 or/6-22 24 randomized controlled trial.pt. 25 controlled clinical trial.pt. 26 randomized.ab. 27 placebo.ab. 28 clinical trials as topic.sh. 29 randomly.ab. 30 trial.ti. 31 or/24-30 32 exp Animals/not humans.sh. 33 31 not 32 34 5 and 23 and 33

* MeSH: Medical Subject Headings; NAFLD: non-alcoholic fatty liver disease.

**Table 2:** Characteristics of included studies.

First Author, Year, Country	Participants	Intervention/Control	Outcome of Interest
Argo, 2015, USA	Steatohepatitis within 6 months.	A: (*n* = 17) n-3 fish oil (1050 mg EPA, 750 mg DHA, 300 mg other omega-3 s each day) for 12 months B: (*n* = 17) placebo (predominantly soybean oil)	Anthropometric assessment Histologic assessment LFT Insulin resistance and lipid profile Abdominal MRI Markers of hepatocyte injury
Boyraz, 2015, Turkey	Obese patients with NAFLD, persistently elevated serum aminotransferase levels	A: (*n* = 56) Diet; 1000 mg of PUFA per day and lifestyle intervention for 12 months B: (*n* = 52) Diet, placebo, and lifestyle intervention	Anthropometric assessment LFT Insulin resistance and lipid profile Liver ultrasonography
Chen, 2008, China	NAFLD patients with abnormal lipid test	A: (*n* = 15) n-3PUFA, low dose (5 g/day) for 6 months B: (*n* = 16) placebo	LFT Lipid profile tests Liver ultrasonography
Dasarathy, 2015, USA	Well-controlled diabetes and NASH patients with NAS ≥ 4 within 6 months	A: (*n* = 18) 2160 mg EPA and 1440 mg DHA daily for 12 months B: (*n* = 19) Placebo (corn oil)	Anthropometric assessment LFT Insulin resistance and lipid profile Histologic assessment
Eriksson, 2018, Sweden	T2DM, MRI showed PDFF > 5.5% (NAFLD) and BMI of 25–40 kg/m^2^.	A: (*n* = 14) OM-3CA 4 g (4 capsules of Epanova [AstraZeneca] each day) for 3 months B: (*n* = 20) matching placebos	Lipid profile Sugar and insulin resistance Plasma levels of DHA, EPA and Oxidative stress biomarkers MRI
Hu, 2015, China	Patients who are pathologically diagnosed NAFLD, aged 20–60 years old	A: (*n* = 26) PUFA of seal oil for (4 g) for 6 months B: (*n* = 17) placebo	LFT Lipid profile and insulin resistance Abdominal ultrasound Histologic assessment
Janczyk, 2015, Poland	(1) Overweight or obese patients aged between 5 and 19 years (2) ALT activity ≥ 1.3 times the ULN (3) Presence of NAFLD by ultrasound or histology	A: (*n* = 30) omega-3 PUFA (DHA and EPA in a 3:2 proportion [450–1300 mg/day]) for 6 months B: (*n* = 34) placebo (sunflower oil)	Anthropometric measurements LFT Lipid profile and insulin resistance Abdominal ultrasound
Kong, 2011, China	NAFLD patients	A: (*n* = 20) PUFA of seal oil for (4 g, composed of EPA, DPA, DHA, each over 25%) for 6 months B: (*n* = 20) placebo (olive oil)	LFT Lipid profile CT of abdomen
Li, 2015, China	Patients pathologically diagnosed with NASH	A: (*n* = 39) PUFA therapy group (daily 50 mL PUFA with 1:1 of EPA and DHA) for 6 months B: (*n* = 39) Normal saline	Anthropometric measurements LFT Lipid profile Histological evaluation
Moulder, 2013 (Abstract), USA	NASH, proven by histology	A: (*n* = 6) a daily omega-3 for 12 months B: (*n* = 7) placebo	NASH biomarkers Insulin and lipid profile tests Erythrocyte fatty acid levels MRI of abdomen Liver biopsy and histological evaluation
Nogueira, 2016, Brazil	People with a proven histological diagnosis of NASH.	A: (*n* = 27) Omega 3 fatty acids (0.315 g per capsules, 3 capsules daily) B: (*n* = 23) Mineral oil (2 mL per capsules)	Liver histopathology, biochemical tests, and anthropometric data
Oscarsson 2018, Sweden	40–75 years old, BMI of 25–40, TG ≥ 150 mg/dL, PDFF-MRI ≥ 5.5%.	A: (*n* = 20) 4 g Epanova capsule (no less than 3400 mg PUFA, contains DPA, DHA, EPA) for 3 months B: (*n* = 21) Placebo	Anthropometric measurements Glucose and insulin resistance Lipid profile LFT MRI of liver
Pacifico, 2015, Italy	Age < 18 years; BMI > 85th percentile elevated ALT level; MRI-diagnosed NAFLD; liver biopsy consistent with NAFLD	A: (*n* = 25) DHA supplementation [250 mg/day] for 6 months B: (*n* = 26) Placebo	Anthropometric measurements Lipid profile and insulin resistance LFT Abdominal MRI Echocardiographic parameters Liver biopsy
Parker, 2019, Australia	Men aged from 18 to 60 years, BMI 25.0~29.9 and WC > 94 cm	A: (*n* = 25) Fish oil group consume 4 × 500 mg capsules (588 mg EPA and 412 mg DHA), for 3 months B: (*n* = 25) placebo group use 4 × 500 mg capsules containing olive oil	Anthropometric assessment Lipid profile LFT Omega-3 Index MRI and proton MRS
Qin, 2015, China	NAFLD patients with hyperlipidemia	A: (n = 36) Fish oil group, 728 mg of EPA and 516 mg of DHA for 3 months B: (n = 34) corn oil, each contained no EPA or DHA	Anthropometric parameters Lipid profile and sugar Insulin resistance LFT, kidney parameters Serum cytokines
Sanyal, 2014, North America	Patients with biopsy-confirmed NASH	A: (*n* = 64) EPA-E 2700 mg/day for 12 months B: (*n* = 55) EPA-E 1800 mg/day for 12 months C: (*n* = 55) Placebo	LFT Serum lipids and insulin resistance Liver biopsy and histological evaluation
Scorletti, 2014, North America	NAFLD patients confirmed by image or histology	A: (*n* = 51) Omacor (4 g/day) contains 1840 mg of EPA and 1520 mg of DHA for 15–18 months B: (*n* = 52) Placebo group uses 4 g olive oil/day	Anthropometric parameters LFT Serum lipid tests and glucose level Fibrosis markers Quantification of erythrocyte enrichment with DHA + EPA MRI of abdomen
Sofi, 2010, Italy	Patients with NAFLD characterized by ultrasonography	A: (*n* = 6) olive oil enriched with n-3 PUFA (830 mg 470 mg EPA and 140 mg DHA) for 12 months B: (*n* = 5) a similar package of olive oil	Anthropometric parameters Physical activity Liver enzymes Serum lipids and insulin resistance Oxidative stress markers Fatty liver evaluation
Song, 2020, China	Adult participants diagnosed fatty liver by ultrasound and were dyslipidemic status	A: (*n* = 21) 450 mg EPA + 1500 mg DHA/day for 3 months B: (*n* = 21) placebo group	Anthropometric measurements Liver enzymes Serum lipids and glucose level CT examination Cytokine determination Serum DHA and EPA concentration analysis
Spadaro, 2008, Italy	NAFLD patients diagnosed by ultrasonography with increased in ALT levels for ≥6 months	A: (*n* = 18) 2 g PUFAs/day plus dietary treatment for 6 months B: (*n* = 18) dietary treatment	Anthropometric measurements Liver enzymes Serum lipids and insulin resistance Ultrasound
Tobin, 2018, USA	Adults with NAFLD and BMI between 18–39.9 kg/m^2^	A: (*n* = 81) 1380 mg EPA and 1140 mg DHA, respectively, for 6 months B: (*n* = 86) placebo as olive oil	Anthropometric data LFT Lipid profile RBC fatty acid content: RBC EPA + DHA, EPA, and DHA Values MRI-PDFF liver fat percentage
Zhu, 2008, China	Patients (age 18–65) with NAFLD with dyslipidemia, elevated liver enzymes	A: (*n* = 66) 2 g n-3 PUFA from seal oils, 3 times a day for 6 months B: (*n*-68) 2 g placebo	Anthropometric data LFT Lipid profile Liver ultrasonography

ALT: alanine aminotransferase; BMI: body mass index; CT: computed tomography; DHA: docosahexaenoic acid; DPA: docosapentaenoic acid; EPA: eicosapentaenoic acid; LFT: liver function test; MRI: magnetic resonance imaging; MRS: magnetic resonance spectroscopy; NAFLD: non-alcoholic fatty liver disease; NAS: NAFLD activity score; NASH: nonalcoholic steatohepatitis; OM-3CA: omega-3 carboxylic acids; PDFF: proton density fat fraction; PUFA: polyunsaturated fatty acid; RBC: red blood cell; T2DM: type 2 diabetes mellitus; ULN: upper limit of normal; WC: waist circumference.

## References

[1] Younossi Z.M., Koenig A.B., Abdelatif D., Fazel Y., Henry L., Wymer M. (2016). Global epidemiology of nonalcoholic fatty liver disease-Meta-analytic assessment of prevalence, incidence, and outcomes. Hepatology.

[2] Disease G.B.D., Injury I., Prevalence C. (2018). Global, regional, and national incidence, prevalence, and years lived with disability for 354 diseases and injuries for 195 countries and territories, 1990–2017: A systematic analysis for the global burden of disease study 2017. Lancet.

[3] Scorletti E., Bhatia L., McCormick K.G., Clough G.F., Nash K., Hodson L., Moyses H.E., Calder P.C., Byrne C.D. (2014). Effects of purified eicosapentaenoic and docosahexaenoic acids in nonalcoholic fatty liver disease: Results from the Welcome* study. Hepatology.

[4] Alkhouri N., McCullough A.J. (2012). Noninvasive diagnosis of NASH and liver fibrosis within the spectrum of NAFLD. Gastroenterol. Hepatol..

[5] McCullough A.J. (2004). The clinical features, diagnosis and natural history of nonalcoholic fatty liver disease. Clin. Liver Dis..

[6] Dulai P.S., Singh S., Patel J., Soni M., Prokop L.J., Younossi Z., Sebastiani G., Ekstedt M., Hagstrom H., Nasr P. (2017). Increased risk of mortality by fibrosis stage in nonalcoholic fatty liver disease: Systematic review and meta-analysis. Hepatology.

[7] Adams L.A., Sanderson S., Lindor K.D., Angulo P. (2005). The histological course of nonalcoholic fatty liver disease: A longitudinal study of 103 patients with sequential liver biopsies. J. Hepatol..

[8] Ekstedt M., Franzen L.E., Mathiesen U.L., Thorelius L., Holmqvist M., Bodemar G., Kechagias S. (2006). Long-term follow-up of patients with NAFLD and elevated liver enzymes. Hepatology.

[9] Pagadala M.R., McCullough A.J. (2012). The relevance of liver histology to predicting clinically meaningful outcomes in nonalcoholic steatohepatitis. Clin. Liver Dis..

[10] Wong V.W., Wong G.L., Choi P.C., Chan A.W., Li M.K., Chan H.Y., Chim A.M., Yu J., Sung J.J., Chan H.L. (2010). Disease progression of non-alcoholic fatty liver disease: A prospective study with paired liver biopsies at 3 years. Gut.

[11] Marchesini G., Brizi M., Bianchi G., Tomassetti S., Bugianesi E., Lenzi M., McCullough A.J., Natale S., Forlani G., Melchionda N. (2001). Nonalcoholic fatty liver disease: A feature of the metabolic syndrome. Diabetes.

[12] Speliotes E.K., Massaro J.M., Hoffmann U., Vasan R.S., Meigs J.B., Sahani D.V., Hirschhorn J.N., O’Donnell C.J., Fox C.S. (2010). Fatty liver is associated with dyslipidemia and dysglycemia independent of visceral fat: The framingham heart study. Hepatology.

[13] Younossi Z.M., Golabi P., de Avila L., Paik J.M., Srishord M., Fukui N., Qiu Y., Burns L., Afendy A., Nader F. (2019). The global epidemiology of NAFLD and NASH in patients with type 2 diabetes: A systematic review and meta-analysis. J. Hepatol..

[14] Mantovani A., Scorletti E., Mosca A., Alisi A., Byrne C.D., Targher G. (2020). Complications, morbidity and mortality of nonalcoholic fatty liver disease. Metabolism.

[15] Rinella M.E. (2015). Nonalcoholic fatty liver disease: A systematic review. JAMA.

[16] Petta S., Gastaldelli A., Rebelos E., Bugianesi E., Messa P., Miele L., Svegliati-Baroni G., Valenti L., Bonino F. (2016). Pathophysiology of non alcoholic fatty liver disease. Int. J. Mol. Sci..

[17] Haas J.T., Francque S., Staels B. (2016). Pathophysiology and mechanisms of nonalcoholic fatty liver disease. Annu. Rev. Physiol..

[18] Imamura F., Micha R., Wu J.H., de Oliveira Otto M.C., Otite F.O., Abioye A.I., Mozaffarian D. (2016). Effects of saturated fat, polyunsaturated fat, monounsaturated fat, and carbohydrate on glucose-insulin homeostasis: A systematic review and meta-analysis of randomised controlled feeding trials. PLoS Med..

[19] Maximos M., Bril F., Portillo Sanchez P., Lomonaco R., Orsak B., Biernacki D., Suman A., Weber M., Cusi K. (2015). The role of liver fat and insulin resistance as determinants of plasma aminotransferase elevation in nonalcoholic fatty liver disease. Hepatology.

[20] Angulo P. (2002). Nonalcoholic fatty liver disease. N. Engl. J. Med..

[21] Ganz M., Szabo G. (2013). Immune and inflammatory pathways in NASH. Hepatol. Int..

[22] Seki E., Schwabe R.F. (2015). Hepatic inflammation and fibrosis: Functional links and key pathways. Hepatology.

[23] Vonghia L., Michielsen P., Francque S. (2013). Immunological mechanisms in the pathophysiology of non-alcoholic steatohepatitis. Int. J. Mol. Sci..

[24] Siriwardhana N., Kalupahana N.S., Moustaid-Moussa N. (2012). Health benefits of n-3 polyunsaturated fatty acids: Eicosapentaenoic acid and docosahexaenoic acid. Adv. Food Nutr. Res..

[25] Dyall S.C. (2015). Long-chain omega-3 fatty acids and the brain: A review of the independent and shared effects of EPA, DPA and DHA. Front. Aging Neurosci..

[26] Shahidi F., Ambigaipalan P. (2018). Omega-3 polyunsaturated fatty acids and their health benefits. Annu. Rev. Food Sci. Technol..

[27] De Roos B., Mavrommatis Y., Brouwer I.A. (2009). Long-chain n-3 polyunsaturated fatty acids: New insights into mechanisms relating to inflammation and coronary heart disease. Br. J. Pharmacol..

[28] Leaf A., Weber P.C. (1988). Cardiovascular effects of n-3 fatty acids. N. Engl. J. Med..

[29] Phillipson B.E., Rothrock D.W., Connor W.E., Harris W.S., Illingworth D.R. (1985). Reduction of plasma lipids, lipoproteins, and apoproteins by dietary fish oils in patients with hypertriglyceridemia. N. Engl. J. Med..

[30] Yang S.J., Chi C.C. (2019). Effects of fish oil supplement on psoriasis: A meta-analysis of randomized controlled trials. BMC Complement. Altern. Med..

[31] Lepretti M., Martucciello S., Burgos Aceves M.A., Putti R., Lionetti L. (2018). Omega-3 fatty acids and insulin resistance: Focus on the regulation of mitochondria and endoplasmic reticulum stress. Nutrients.

[32] Pahlavani M., Ramalho T., Koboziev I., LeMieux M.J., Jayarathne S., Ramalingam L., Filgueiras L.R., Moustaid-Moussa N. (2017). Adipose tissue inflammation in insulin resistance: Review of mechanisms mediating anti-inflammatory effects of omega-3 polyunsaturated fatty acids. J. Investig. Med..

[33] Chalasani N., Younossi Z., Lavine J.E., Diehl A.M., Brunt E.M., Cusi K., Charlton M., Sanyal A.J. (2012). The diagnosis and management of non-alcoholic fatty liver disease: Practice Guideline by the American association for the study of liver diseases, American College of gastroenterology, and the American gastroenterological association. Hepatology.

[34] European Association for the Study of the Liver, European Association for the Study of Diabetes, European Association for the Study of Obesity (2016). EASL-EASD-EASO Clinical Practice Guidelines for the management of non-alcoholic fatty liver disease. J. Hepatol..

[35] Moher D., Liberati A., Tetzlaff J., Altman D.G., Group P. (2009). Preferred reporting items for systematic reviews and meta-analyses: The PRISMA statement. Open Med..

[36] Follmann D., Elliott P., Suh I., Cutler J. (1992). Variance imputation for overviews of clinical trials with continuous response. J. Clin. Epidemiol..

[37] Higgins J.P., Green S. (2011). Cochrane Handbook for Systematic Reviews of Interventions.

[38] Higgins J.P., Altman D.G., Gøtzsche P.C., Jüni P., Moher D., Oxman A.D., Savovic J., Schulz K.F., Cochrane Bias Methods Group, Cochrane Statistical Methods Group (2011). The Cochrane Collaboration’s tool for assessing risk of bias in randomised trials. BMJ.

[39] AlGhamdi K., Khurrum H. (2013). Methotrexate for the treatment of generalized vitiligo. Saudi. Pharm. J..

[40] Argo C.K., Patrie J.T., Lackner C., Henry T.D., De Lange E.E., Weltman A.L., Shah N.L., Al-Osaimi A.M., Pramoonjago P., Jayakumar S. (2015). Effects of n-3 fish oil on metabolic and histological parameters in NASH: A double-blind, randomized, placebo-controlled trial. J. Hepatol..

[41] Boyraz M., Pirgon Ö., Dündar B., Çekmez F., Hatipoğlu N. (2015). Long-term treatment with n-3 polyunsaturated fatty acids as a monotherapy in children with nonalcoholic fatty liver disease. J. Clin. Res. Pediatric Endocrinol..

[42] Dasarathy S., Dasarathy J., Khiyami A., Yerian L., Hawkins C., Sargent R., McCullough A.J. (2015). Double-blind randomized placebo-controlled clinical trial of omega 3 fatty acids for the treatment of diabetic patients with nonalcoholic steatohepatitis. J. Clin. Gastroenterol..

[43] Eriksson J.W., Lundkvist P., Jansson P.A., Johansson L., Kvarnström M., Moris L., Miliotis T., Forsberg G.B., Risérus U., Lind L. (2018). Effects of dapagliflozin and n-3 carboxylic acids on non-alcoholic fatty liver disease in people with type 2 diabetes: A double-blind randomised placebo-controlled study. Diabetologia.

[44] Janczyk W., Lebensztejn D., Wierzbicka-Rucinska A., Mazur A., Neuhoff-Murawska J., Matusik P., Socha P. (2015). Omega-3 fatty acids therapy in children with nonalcoholic fatty liver disease: A randomized controlled trial. J. Pediatrics.

[45] Li Y.H., Yang L.H., Sha K.H., Liu T.G., Zhang L.G., Liu X.X. (2016). Efficacy of poly-unsaturated fatty acid therapy on patients with nonalcoholic steatohepatitis. World J. Gastroenterol..

[46] Moulder G., Smith E.Z., Caldwell S.H., Argo C.K. (2013). RBC lipid composition is favorably altered in NASH patients treated with omega-3 fish oil versus placebo. Hepatology.

[47] Nogueira M.A., Oliveira C.P., Ferreira Alves V.A., Stefano J.T., Rodrigues L.S., Torrinhas R.S., Cogliati B., Barbeiro H., Carrilho F.J., Waitzberg D.L. (2016). Omega-3 polyunsaturated fatty acids in treating non-alcoholic steatohepatitis: A randomized, double-blind, placebo-controlled trial. Clin. Nutr..

[48] Oscarsson J., Önnerhag K., Risérus U., Sundén M., Johansson L., Jansson P.A., Moris L., Nilsson P.M., Eriksson J.W., Lind L. (2018). Effects of free omega-3 carboxylic acids and fenofibrate on liver fat content in patients with hypertriglyceridemia and non-alcoholic fatty liver disease: A double-blind, randomized, placebo-controlled study. J. Clin. Lipidol..

[49] Pacifico L., Bonci E., Di Martino M., Versacci P., Andreoli G., Silvestri L.M., Chiesa C. (2015). A double-blind, placebo-controlled randomized trial to evaluate the efficacy of docosahexaenoic acid supplementation on hepatic fat and associated cardiovascular risk factors in overweight children with nonalcoholic fatty liver disease. Nutr. Metab. Cardiovasc. Dis. NMCD.

[50] Parker H.M., Cohn J.S., O’Connor H.T., Garg M.L., Caterson I.D., George J., Johnson N.A. (2019). Effect of fish oil supplementation on hepatic and visceral fat in overweight men: A randomized controlled trial. Nutrients.

[51] Qin Y., Zhou Y., Chen S.H., Zhao X.L., Ran L., Zeng X.L., Wu Y., Chen J.L., Kang C., Shu F.R. (2015). Fish oil supplements lower serum lipids and glucose in correlation with a reduction in plasma fibroblast growth factor 21 and prostaglandin E2 in nonalcoholic fatty liver disease associated with hyperlipidemia: A randomized clinical trial. PLoS ONE.

[52] Chen R., Guo Q., Zhu W.J., Xie Q., Wang H., Cai W. (2008). Therapeutic efficacy of omega-3 polyunsaturated fatty acid capsule in treatment of patients with non-alcoholic fatty liver disease. World Chin. J. Dig..

[53] Sanyal A.J., Abdelmalek M.F., Suzuki A., Cummings O.W., Chojkier M. (2014). No significant effects of ethyl-eicosapentanoic acid on histologic features of nonalcoholic steatohepatitis in a phase 2 trial. Gastroenterology.

[54] Sofi F., Giangrandi I., Cesari F., Corsani I., Abbate R., Gensini G.F., Casini A. (2010). Effects of a 1-year dietary intervention with n-3 polyunsaturated fatty acid-enriched olive oil on non-alcoholic fatty liver disease patients: A preliminary study. Int. J. Food Sci. Nutr..

[55] Song L., Zhao X., Ouyang P., Guan Q., Yang L., Peng F., Du H., Yin F., Yan W., Yu W. (2020). Combined effect of omega-3 fatty acids and phytosterol ester on alleviating hepatic steatosis in NAFLD subjects: A double-blind placebo-controlled clinical trial. Br. J. Nutr..

[56] Spadaro L., Magliocco O., Spampinato D., Piro S., Oliveri C., Alagona C., Papa G., Rabuazzo A.M., Purrello F. (2008). Effects of n-3 polyunsaturated fatty acids in subjects with nonalcoholic fatty liver disease. Dig. Liver Dis..

[57] Tobin D., Brevik-Andersen M., Qin Y., Innes J.K., Calder P.C. (2018). Evaluation of a high concentrate omega-3 for correcting the omega-3 fatty acid nutritional deficiency in non-alcoholic fatty liver disease (CONDIN). Nutrients.

[58] Kong X. (2011). A Basic and Clinical Reseach of ω-3 Polyunsaturated Fatty Acids to Seal Non-alcoholic Fatty Liver Disease. Master‘s Thesis.

[59] Zhu F.S., Liu S., Chen X.M., Huang Z.G., Zhang D.W. (2008). Effects of n-3 polyunsaturated fatty acids from seal oils on nonalcoholic fatty liver disease associated with hyperlipidemia. World J. Gastroenterol..

[60] Hu Z.W. (2015). Therapeutic effect of seal oil on non-alcoholic fatty liver disease. Acad. J. Guangzhou Med. Univ..

[61] Hernaez R., Lazo M., Bonekamp S., Kamel I., Brancati F.L., Guallar E., Clark J.M. (2011). Diagnostic accuracy and reliability of ultrasonography for the detection of fatty liver: A meta-analysis. Hepatology.

[62] Saadeh S., Younossi Z.M., Remer E.M., Gramlich T., Ong J.P., Hurley M., Mullen K.D., Cooper J.N., Sheridan M.J. (2002). The utility of radiological imaging in nonalcoholic fatty liver disease. Gastroenterology.

[63] Guido M., Rugge M. (2004). Liver biopsy sampling in chronic viral hepatitis. Semin. Liver Dis..

[64] Tannapfel A., Denk H., Dienes H.P., Langner C., Schirmacher P., Trauner M., Flott-Rahmel B. (2010). Histopathological diagnose of non-alcoholic and alcoholic fatty liver disease. Z. Gastroenterol..

[65] Marsman H.A., Heger M., Kloek J.J., Nienhuis S.L., van Werven J.R., Nederveen A.J., Ten Kate F.J., Stoker J., van Gulik T.M. (2011). Reversal of hepatic steatosis by omega-3 fatty acids measured non-invasively by (1) H-magnetic resonance spectroscopy in a rat model. J. Gastroenterol. Hepatol..

[66] Parker H.M., Johnson N.A., Burdon C.A., Cohn J.S., O’Connor H.T., George J. (2012). Omega-3 supplementation and non-alcoholic fatty liver disease: A systematic review and meta-analysis. J. Hepatol..

[67] Yu L., Yuan M., Wang L. (2017). The effect of omega-3 unsaturated fatty acids on non-alcoholic fatty liver disease: A systematic review and meta-analysis of RCTs. Pak. J. Med. Sci..

[68] Zivkovic A.M., German J.B., Sanyal A.J. (2007). Comparative review of diets for the metabolic syndrome: Implications for nonalcoholic fatty liver disease. Am. J. Clin. Nutr..

[69] Calder P.C. (2013). Omega-3 polyunsaturated fatty acids and inflammatory processes: Nutrition or pharmacology?. Br. J. Clin. Pharmacol..

[70] Wall R., Ross R.P., Fitzgerald G.F., Stanton C. (2010). Fatty acids from fish: The anti-inflammatory potential of long-chain omega-3 fatty acids. Nutr. Rev..

[71] Skulas-Ray A.C., Wilson P.W.F., Harris W.S., Brinton E.A., Kris-Etherton P.M., Richter C.K., Jacobson T.A., Engler M.B., Miller M., Robinson J.G. (2019). Omega-3 fatty acids for the management of hypertriglyceridemia: A science advisory from the American Heart Association. Circulation.

